# Characterization of Some Dermato-Cosmetic Preparations with Marine Lipids from Black Sea Wild Stingray

**DOI:** 10.3390/md21070408

**Published:** 2023-07-19

**Authors:** Magdalena Mititelu, Monica Licu, Carmen Elena Lupu, Sorinel Marius Neacșu, Gabriel Olteanu, Stanciu Gabriela, Doina Drăgănescu, Carmen-Nicoleta Oancea, Ștefan Sebastian Busnatu, Lucian Hîncu, Maria Viorica Ciocîlteu, Dumitru Lupuleasa

**Affiliations:** 1 Department of Clinical Laboratory and Food Safety, Faculty of Pharmacy, University of Medicine and Pharmacy Carol Davila, 020956 Bucharest, Romania; magdalena.mititelu@umfcd.ro; 2 Department of Medical Psychology, Faculty of Medicine, “Carol Davila” University of Medicine and Pharmacy, 050474 Bucharest, Romania; monica.licu@umfcd.ro; 3 Department of Mathematics and Informatics, Faculty of Pharmacy, “Ovidius” University of Constanta, 6 Capitan Aviator Al. Serbanescu Street, Campus, C Block, 900001 Constanta, Romania; clupu@univ-ovidius.ro; 4 Department of Pharmaceutical Technology and Bio-pharmacy, Faculty of Pharmacy, Carol Davila University of Medicine and Pharmacy, 020945 Bucharest, Romania; dumitru.lupuliasa@umfcd.ro; 5 Department of Chemistry and Chemical Engineering, Ovidius University of Constanta, 900527 Constanta, Romania; 6 Department of Pharmaceutical and Computer Physics, Faculty of Pharmacy, Carol Davila University of Medicine and Pharmacy, 020956 Bucharest, Romania; doina.draganescu@umfcd.ro; 7 Department of Biochemistry, Faculty of Medicine, University of Medicine and Pharmacy from Craiova, 200345 Craiova, Romania; carmen.oancea@umfcv.ro; 8 Department of Cardio-Thoracic Pathology, Faculty of Medicine, “Carol Davila” University of Medicine and Pharmacy, 050474 Bucharest, Romania; stefan.busnatu@umfcd.ro; 9 Department of Drug Industry and Pharmaceutical Biotechnologies Department, Faculty of Pharmacy, University of Medicine and Pharmacy Carol Davila, 020956 Bucharest, Romania; lucian.hincu@umfcd.ro; 10 Department of Analytical and Instrumental Chemistry, Faculty of Pharmacy, University of Medicine and Pharmacy of Craiova, Petru Rares Street, no. 2–4, 200638 Craiova, Romania; maria.ciocilteu@umfcv.ro

**Keywords:** healing effect, polyunsaturated fatty acids, stingray liver oil, fatty ointment, emulgels, anti-inflammatory effect

## Abstract

The traditional knowledge about the therapeutic and nutritional value of fish has been unanimously recognized among the population since ancient times. So, thanks to the therapeutic virtues of these marine animals, it was possible to develop therapies for certain pathologies as well as the use of bioactive compounds as adjunctive therapies incorporated into the treatment regimen of patients. In the present study, stingray liver oil from wild species collected from the Romanian coast of the Black Sea was isolated and analyzed. Fatty acid analysis was performed by gas chromatography. The analysis of the distribution of fatty acids in the composition of stingray liver oil indicates a ratio of 2.83 of omega 3 fatty acids to omega 6, a ratio of 1.33 of polyunsaturated fatty acids to monounsaturated fatty acids, an iodine index of 111.85, and a total percentage of 68.98% of unsaturated fatty acids. Stingray liver oil was used to evaluate the healing action after preparing a fatty ointment. According to the experimental data, a complete regeneration capacity of the wounds was noted in 12 days without visible signs. Four emulgels with stingray liver oil were formulated and analyzed from a rheological and structural point of view in order to select the optimal composition, after which the anti-inflammatory effect on inflammation caused in laboratory rats was studied and an anti-inflammatory effect was found significant (a maximum inhibitory effect of 66.47% on the edemas induced by the 10% kaolin suspension and 65.64% on the edemas induced by the 6% dextran solution).

## 1. Introduction

The common stingray (*Dasyatis pastinaca*) is a member of the class Chondrichthyes (which includes mostly large cartilaginous fish and prey fish) and is related to the shark. Cartilaginous fish are also among the oldest vertebrates and possess a complex digestive system [1].

Stingray liver oil is particularly valuable due to its high content of monounsaturated and polyunsaturated fatty acids. The main ingredients in stingray liver oil are monounsaturated fatty acids, omega-3 fatty acids, important amounts of docosahexaenoic acid (DHA) and eicosapentanoic acid (EPA), vitamin A, vitamin E, vitamin D, and minerals. The common stingray (*Dasyatis pastinaca*) is a cartilaginous fish related to the shark. It lives along shores with warmer water. It is also found in the Black Sea, approaching the coast when the water reaches 12 °C, looking for a place on the sandy bottoms, and retreating to the depths when the water cools. The weapon of a stingray is represented by one or more sharp spikes located at the end of the tail, through which it spreads a dangerous poison when it stings the victim. It feeds on small fish, mollusks, and crustaceans. The meat of stingrays is not eaten. The liver represents approximately 23% of the female and 11% of the male’s body weight and contains 52–70% fat. A quality oil is extracted from it, rich in anti-rickets vitamins (A, D, and E) and polyunsaturated fatty acids (omega 3, 6, 9, etc.), also used in the healing of external wounds [2,3,4].

In the specialized literature, there are limited studies on *Dasyatis pastinaca*, although the therapeutic effects of the liver of marine animals are multiple and indisputable [5,6,7,8,9,10,11,12]. Lipid fractions isolated from *Dasyatis zugei* with petroleum ether and diethyl ether appear to have analgesic and anti-inflammatory properties highlighted in animal model studies [13]. *Dasyatis jenkinsii*, also known as the sharp-nosed stingray, belongs to the class *Elasmobranchii* and has therapeutic virtues in the treatment of inflammatory diseases and arthritis [14].

Stingray skin is a growing trend today; most of the big fashion designers use it to make accessories that delight fashion and luxury lovers all over the world. In the fashion industry, stingray skin is considered one of the most unique materials. With its special texture, stingray skin gives any fashion product a special quality, easily standing out in the crowd. In addition to the visual appeal that this leather possesses, accessories made of stingray leather are more resistant to abrasion, scratches, and high temperatures that could otherwise compromise some leather products. In the past, stingray skin was used for the handles of swords and tools because it aided grip and was resistant to damage from moisture or sweat. In the 21st century, stingray skin enjoys popularity among fashion lovers, not only because of its attractive design but also because of the superior quality of the product itself [15].

Depending on the width, stingray skin can be used for wallets, watch straps or other small accessories, belts, handbags, shoes, and other Western clothing items, interior decoration, automobiles, or other upholstery [15].

Marine animals represent reservoirs of bioactive compounds with impressive therapeutic virtues [16,17,18]. Fish liver oil, rich in polyunsaturated fatty acids, presents real health benefits, as reported throughout history in the specialized literature [19,20,21,22,23,24,25].

The effect of fatty acids in fish oil is not only limited internally, through food intake or the administration of supplements, but also externally, through skin applications. Their application in skin diseases includes photoaging, dermatitis, cancer, healing (scarring properties), and melanogenesis [26,27,28,29].

Burns, skin wounds, chronic wounds, and ulcers have affected and continue to affect millions of people around the world. The processes at the cellular and molecular level that occur in the inflammatory phase of wound healing are initiated and greatly amplified by pro-inflammatory cytokines, whose synthesis and activity can be modulated by polyunsaturated fatty acids [30,31]. Fatty acids have been shown to be essential in skin tissue reconstruction processes [32]. Animal studies have demonstrated the beneficial effect of the fatty acids present in cod liver oil on the prevention or healing of skin wounds, both topically and internally (orally as a supplement or by injection) [33,34,35,36,37,38,39,40,41,42]. Fish liver oil stimulates angiogenesis, thus promoting wound healing. Compared to olive oil, fish liver oil has faster tissue repair activity [33].

The utilization of bioactive compounds from marine species for therapeutic purposes must also take into account the harvesting areas because the marine environment can be degraded in certain regions due to the increased level of pollution [43]. Various contaminants from the marine environment (heavy metals, pesticides, and microplastics) can also be found in marine organisms in concentrations that may endanger the safety of the consumer [44,45].

The purpose of this study is the characterization and use of stingray liver oil for the production of dermato-cosmetic preparations (ointments and emulgels) and the analysis of the healing and anti-inflammatory effects on laboratory animals. Stingray liver oil is a less studied marine oil that can be exploited as a valuable bioactive by-product resulting from the processing of stingray specimens exploited on farms to obtain leather products.

## 2. Results

### 2.1. Characteristics of Stingray Liver Oil

Table 1 shows the main physico-chemical properties of the obtained stingray liver oil. The oil is in the form of a clear, oily liquid with a characteristic odor and orange-reddish color.

**Figure 1:**
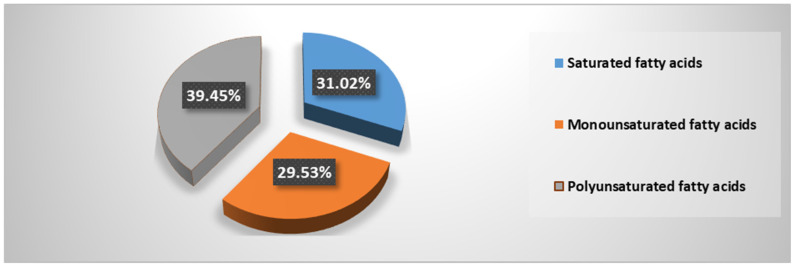
Distribution of different fractions of fatty acids in stingray liver oil.

Table 2 and Figure 1 show the distribution of fatty acids in the extracted and analyzed stingray liver oil. The predominant fraction is represented by polyunsaturated fatty acids (39.45%), while the total of unsaturated fatty acids represents approximately 69%, which explains the high iodine index (high degree of unsaturation of the analyzed oil).

### 2.2. Characteristics of Stingray Liver Oil Ointment

**Figure 2:**
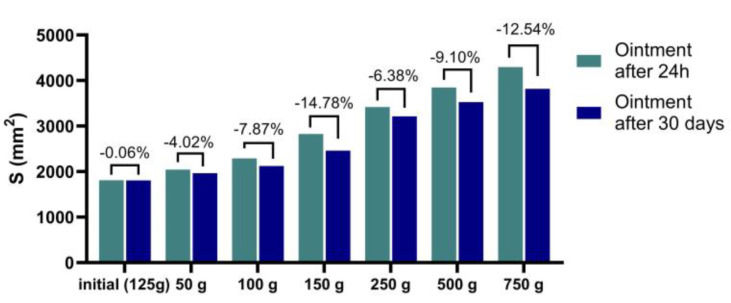
The stretching surface depending on the applied mass.

The results obtained from the analysis of the fatty ointment with stingray liver oil are shown in Table 3 and Figure 2. The ointment is homogeneous, stable, has a pleasant effect when applied to the skin, and has a pH compatible with the skin.

In addition, the ointment shows high extension values immediately after preparation, proof that it has a suitable consistency and a high stretching capacity. Over time, the creams maintain their consistency and stretchability well (Figure 2).

### 2.3. Evaluation of the Healing Action of Stingray Liver Oil Ointment

**Figure 3:**
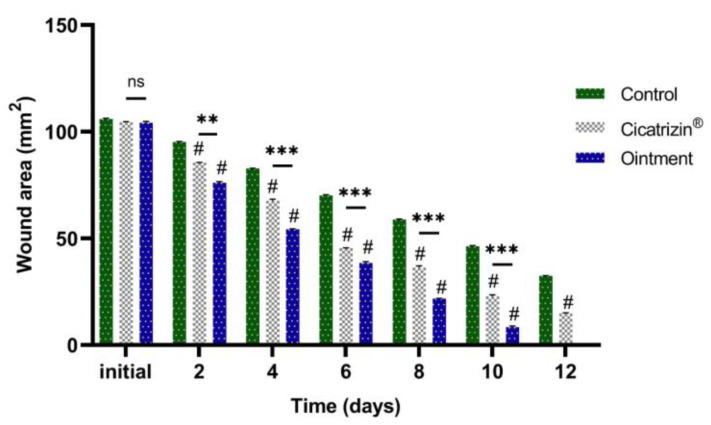
Evolution of the wound surface: average wound area (mm^2^) ± SD (standard deviation). The data were represented as mean ± SD and analyzed using ANOVA. # *p* < 0.001 has statistical significance versus the control group, while ** *p* < 0.01 and *** *p* < 0.001 refer to statistical significance between the group treated with the stingray liver oil ointment and the group treated with Cicatrizin^®^.

The evolution of wound healing is presented in Figure 3. According to the experimental data, an accelerated healing process can be noted in the group of animals treated with the stingray liver oil ointment.

All treatment groups were compared to the control group, to each other (treated with the stingray liver oil ointment and treated with Cicatrizin^®^), and to each other between treatments (days).

No statistical differences were found between the three groups at the initial moment. In the control group, there was a statistically significant decrease (*p* ≤ 0.001) between days 4 and 10. A significant decrease (*p* ≤ 0.001) in the group treated with the stingray liver oil ointment and the group treated with Cicatrizin^®^ was seen on Day 2.

**Figure 4:**
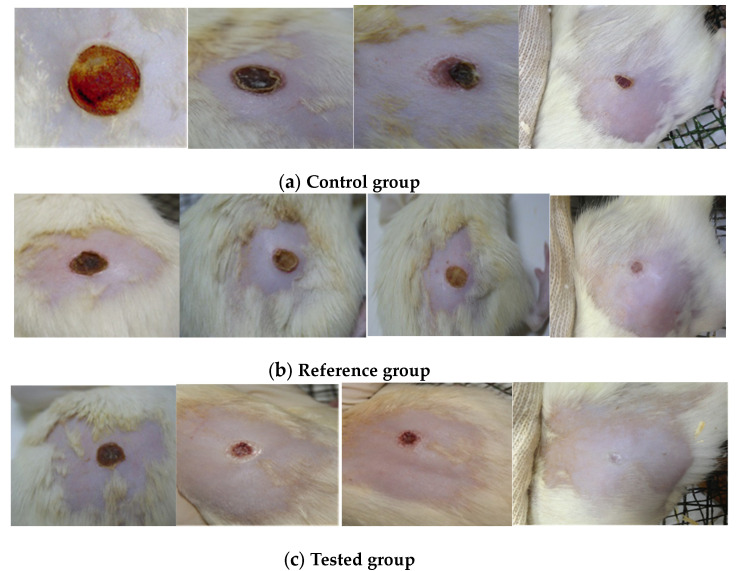
The evolution of the wounds at different times (initially, after 2 days, after 4 days, and after 8 days): (**a**) the untreated group (control); (**b**) the group treated with Cicatrizin^®^ (reference); (**c**) the group treated with stingray liver oil ointment (tested).

According to the data presented in Figure 4, a strong healing effect can be noted in the case of the treatment with stingray liver oil ointment (tested group), with a good evolution over time, significantly increased compared to the wound healing process in the untreated control group, and clearly superior to the reference group treated with Cicatrizin^®^.

**Figure 5:**
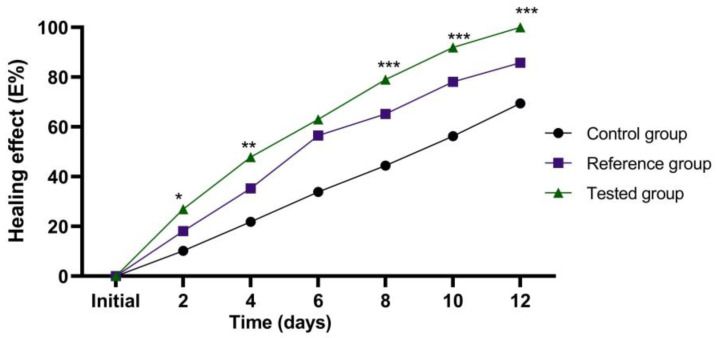
The evolution of the healing effect (E%) during the treatment. * *p* < 0.05, ** *p* < 0.01, and *** *p* < 0.001 refer to statistical significance between the reference group and the tested group.

After 8 days of treatment, a cure of 79.08% can be noted in the case of animals treated with the prepared ointment, 65.23% in the case of animals treated with Cicatrizin^®^, and 44.44% in the case of untreated animals (Figure 5).

The post hoc analysis revealed a greater difference between Days 2 and 12 assessed in the study between the tested group and the control group.

### 2.4. Characteristics of Catfish Liver Oil Emulgels

**Figure 6:**
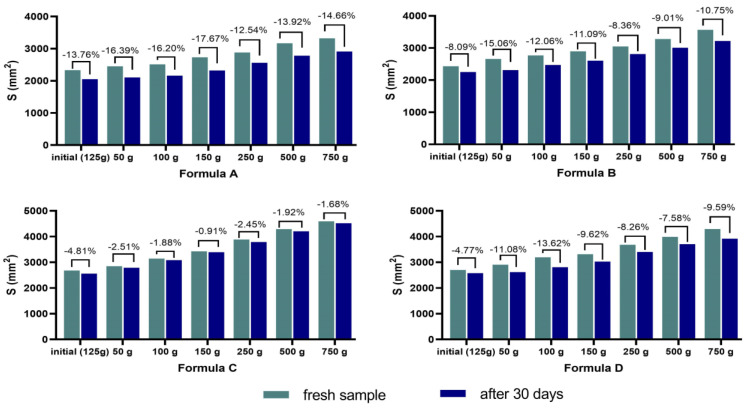
Spreading area for the emulgels prepared: Formula A, Formula B, Formula C and Formula D.

The results obtained from the analysis of the emulgels with stingray liver oil are shown in Table 4 and Figure 6. According to the results obtained, the prepared formulas are stable and homogeneous (Table 4), but the best spreading surface was noted for Formula C (Figure 6), with good maintenance over time.

**Figure 7:**
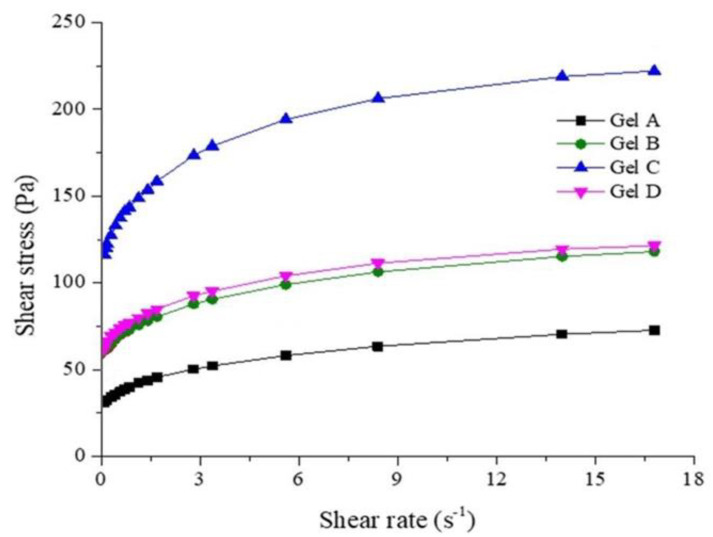
The rheograms for emulgels analyzed at 33 °C.

The increase in shear stress with the shear rate indicates a non-Newtonian behavior of the emulgels (Figure 7).

The rheological characteristics of the prepared emulgels are presented in Table 5 and Table 6. Table 5 shows the flow parameters and viscosity of the analyzed emulgels.

**Figure 8:**
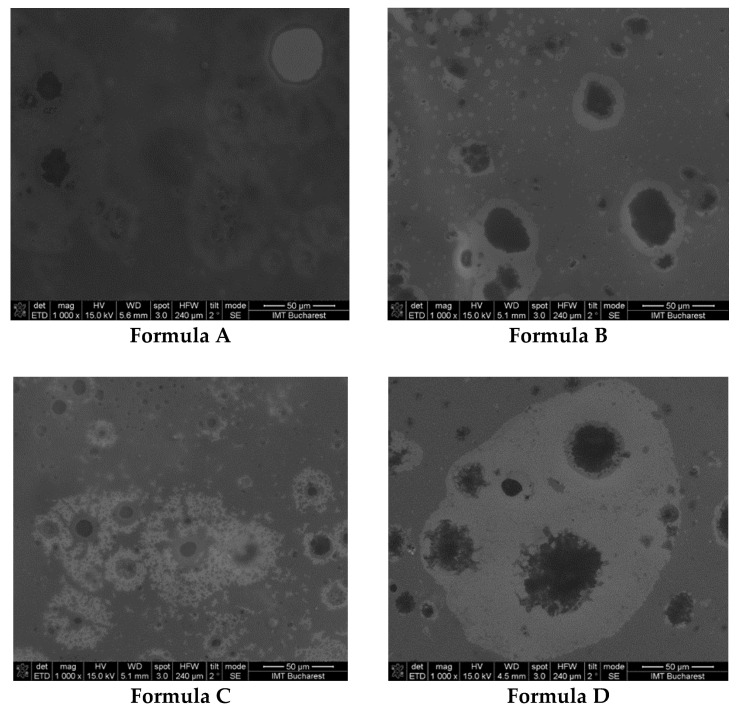
SEM images from the emulgels prepared: Formula A, Formula B, Formula C and Formula D.

The internal morphology of the emulgels is presented in Figure 8. According to the results of the rheological and morphological analysis, the optimal formula for emulsifying stingray liver oil in a gel base is Formula C.

### 2.5. Evaluation of the Anti-Inflammatory Action of Stingray Liver Emulgel (Formula C)

**Figure 9:**
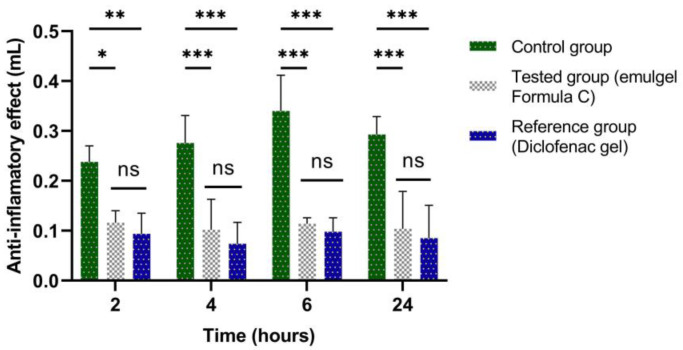
The anti-inflammatory effect of stingray liver oil emulgel on inflammatory edema induced with 10% kaolin suspension. The data were represented as mean ± SD and analyzed using ANOVA. * *p* < 0.05, ** *p* < 0.01, and *** *p* < 0.001 versus the control group, while ns: *p* > 0.05 statistical significance between the reference group and the tested group.

**Figure 10:**
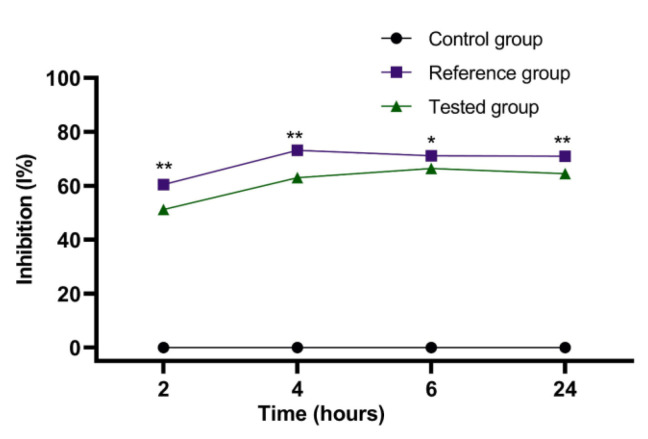
The evolution of the inhibition effect (I%) on inflammatory edema induced with 10% kaolin suspension during the treatment. * *p* < 0.05 and ** *p* < 0.01 refer to statistical significance between the reference group and the tested group.

**Figure 11:**
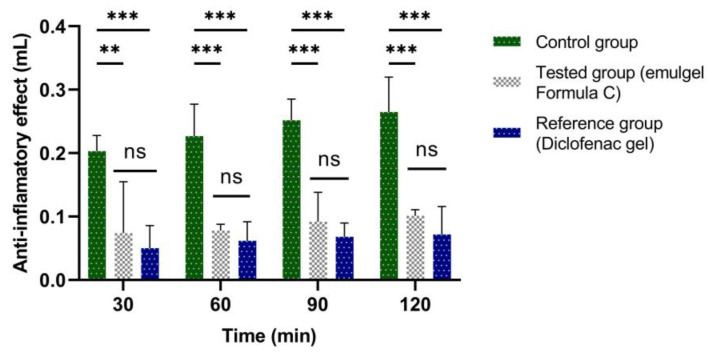
The anti-inflammatory effect of stingray liver oil emulgel on inflammatory edema induced with a 6% dextran solution. The data were represented as mean ± SD and analyzed using ANOVA. ** *p* < 0.01, and *** *p* < 0.001 versus the control group, while ns: *p* > 0.05 statistical significance between the reference group and the tested group.

**Figure 12:**
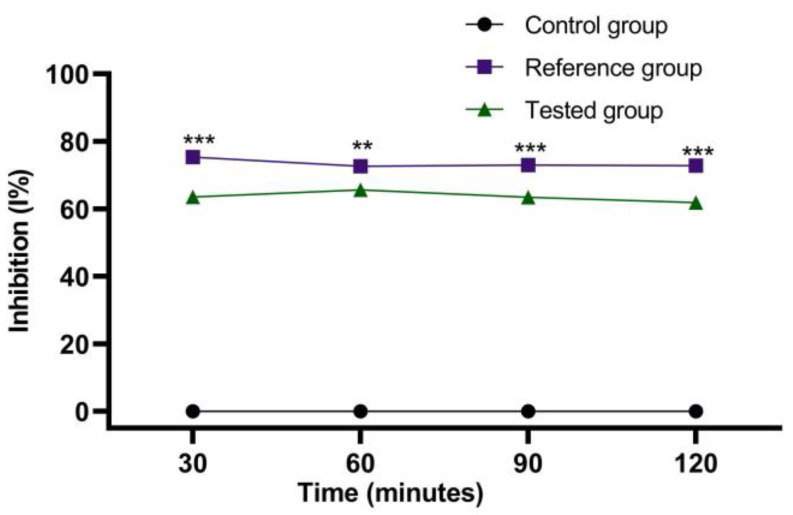
The evolution of the inhibition effect (I%) on inflammatory edema induced with a 6% dextran solution during the treatment. ** *p* < 0.01 and *** *p* < 0.001 refer to statistical significance between the reference group and the tested group.

The experimental results of the evaluations of the anti-inflammatory effect carried out on the emulgel prepared according to Formula C (Table 4) are presented in Figure 9, Figure 10, Figure 11 and Figure 12. They show the evolution of the edema inhibition effect (I%) over time for the preparations used (emulgel Formula C and Diclofenac gel) compared to the untreated control group.

The anti-inflammatory effect of stingray liver oil emulgel on inflammatory edema is significantly different from the control group but not from the reference group (Figure 9 and Figure 10).

It can be noted from Figure 10 that there is a significant anti-inflammatory effect for the stingray liver oil emulgel, with the edema inhibition effect being between 51.26% and 66.47% on the edemas induced by the 10% kaolin suspension. The inhibitory effect of Diclofenac gel was between 60.5% and 73.19% and exhibited a maximum percent at a time period of 4 h.

In the case of edemas induced by the 6% dextran solution (Figure 12), the edema inhibition effect for emulgel was also significant, between 61.89% and 65.64%, while for Diclofenac gel chosen as a reference, the inhibitory effect was between 72.69% and 75.37%.

## 3. Discussion

The extraction technique of stingray liver oil was not aimed at obtaining a high extraction yield but at the application of a method that preserves the bioactive principles of the lipid composition and does not contaminate the final lipid fraction with toxic solvents in the event that the oil is also used for oral administration. About 260 g of the final oily fraction was extracted from 500 g of stingray liver. The value of the peroxide index (Table 1) indicates reduced oxidative degradation during the extraction process. The main valuable components of the lipid fraction are unsaturated fatty acids (especially omega 3 and omega 6 essential fatty acids), fat-soluble vitamins (A, D, and E), compounds with increased therapeutic potential, and compounds that are easily oxidizable. That is why it is recommended to condition the oil in hermetically sealed brown glass bottles in a cool (8–15 °C) and dark place.

The results from the analysis of fatty acids in the composition of stingray liver oil indicate a predominance of unsaturated fatty acids (Figure 1), as well as a predominance of omega 3 fatty acids compared to omega 6 fatty acids (Table 2). As with other lipids of marine origin, such as cod liver oil, salmon liver oil, and total lipid extract from mussels, the results indicate an increased amount of polyunsaturated fatty acids and a super-unit ratio of omega 3 to omega 6 acids (characteristic of marine fats) [46,47].

Omega 3 fatty acids, which are anti-inflammatory, unsaturated fatty acids, and fat-soluble vitamins in the composition of the oil have an emollient, restructuring, and antioxidant effect on the epidermis. There are clinical studies that indicate fish oil generally has regenerative, anti-inflammatory, and protective effects on the epidermis. Positive effects on certain dermatological conditions (dermatitis, eczema) were noted in the case of marine lipids [48,49,50,51].

Treatment of animals with stingray liver oil healing ointment resulted in complete healing in all animals after 12 days of treatment (Figure 3). On the 6th day of treatment, the primary crust formed in the region of the wounds began to detach. After eight days, the crust was completely detached in all treated animals, and the remaining wound was covered with fine granular tissue (Figure 4C). After 10 days, the healing finished almost completely without very visible signs. Through the complex chemical composition presented by the healing ointment with stingray liver oil, it heals wounds on the skin and reduces inflammation. Healing and regeneration of the damaged tissues were achieved quickly and almost completely after only 10 days without obvious signs.

The cicatrizing ointment with stingray liver oil used in the study was well tolerated by the skin and presented a cicatrizing action on the experimental lesions produced in rats that was more effective than that of the Cicatrizin^®^ ointment taken as a reference. Cicatrizin^®^ ointment was chosen because it has a complex composition and contains natural plant extracts that are recognized for their healing effects. In the case of animals treated with Cicatrizin^®^ ointment, a fine, dry crust was formed, which completely detached after 10 days of treatment, and healing occurred after 13–14 days. After the regeneration process, obvious traces remained. In the control group, the healing of the wounds was much more difficult, and the remaining marks were much more obvious, with the tissue under the crust remaining inflamed for a long time. The evolution of the healing effect over time indicates significant differences between the groups studied since the first administrations (Figure 5); thus, after six days, the wounds of the group tested with ointment had healed by 63.05%, the control group had healed by 33.87%, and the reference group had healed by 56.51%. After ten days of treatment, the percentage of wound healing was 91.94% in the group tested with ointment, 78.16% in the reference group treated with Cicatrizin^®^, and only 56.32% in the untreated control group.

The rheological characteristics of emulgels made with stingray liver oil depend on the concentration of the lipid fraction emulsified in the gel base. The formula with the best rheological parameters and characteristics (spreading capacity, flow parameters, plasticity, uniformity of distribution of emulsified particles) is Formula C. That is why Formula C was chosen to test its anti-inflammatory action.

The stingray liver oil proved to have a significant anti-inflammatory effect with a maximum at six hours of treatment in the case of edemas induced by the 10% kaolin suspension (66.47%) compared to the Diclofenac gel chosen as a reference, which registered a maximum effect after four hours of treatment at 73.19% (Figure 10). In the case of edema induced by the 6% dextran solution (Figure 12), the maximum effect of the emulgel with stingray liver oil was recorded after 60 minutes of treatment (65.64%), while with the reference gel, the maximum effect was recorded after 30 minutes of treatment (75.37%).

Along with vegetable products that represent important sources of antioxidants and fibers [52,53], ingredients with beneficial effects in the metabolic syndrome and especially in cardiovascular diseases, marine lipids are also important sources of antioxidants and polyunsaturated fatty acids with beneficial effects in many diseases, including the metabolic syndrome and cardiovascular diseases [54,55]. As a result, it is recommended to capitalize on the therapeutic potential of marine lipids both in medicinal formulas for internal and external use.

## 4. Materials and Methods

### 4.1. Extraction of Black Sea Stingray (Dasyatis pastinaca) Liver Oil

The laboratory technology used for the extraction of stingray liver oil sought to obtain a high-quality oil without toxic impurities from potentially toxic solvents and without the use of high temperatures that degrade the valuable compounds in the oil (polyunsaturated fatty acids, fat-soluble vitamins, etc.).

**Figure 13:**
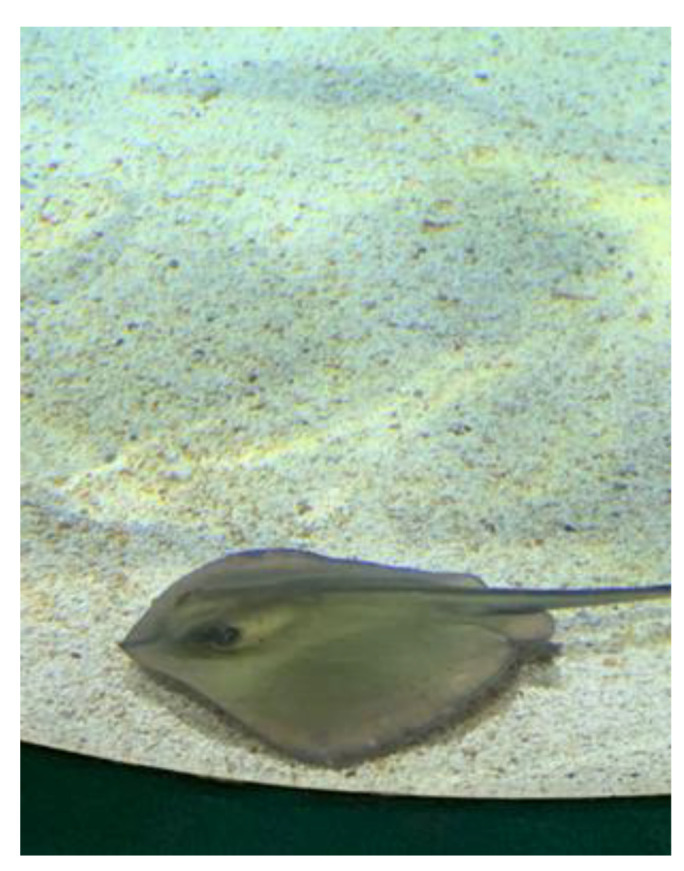
Black Sea common stingray (*Dasyatis pastinaca*).

**Figure 14:**
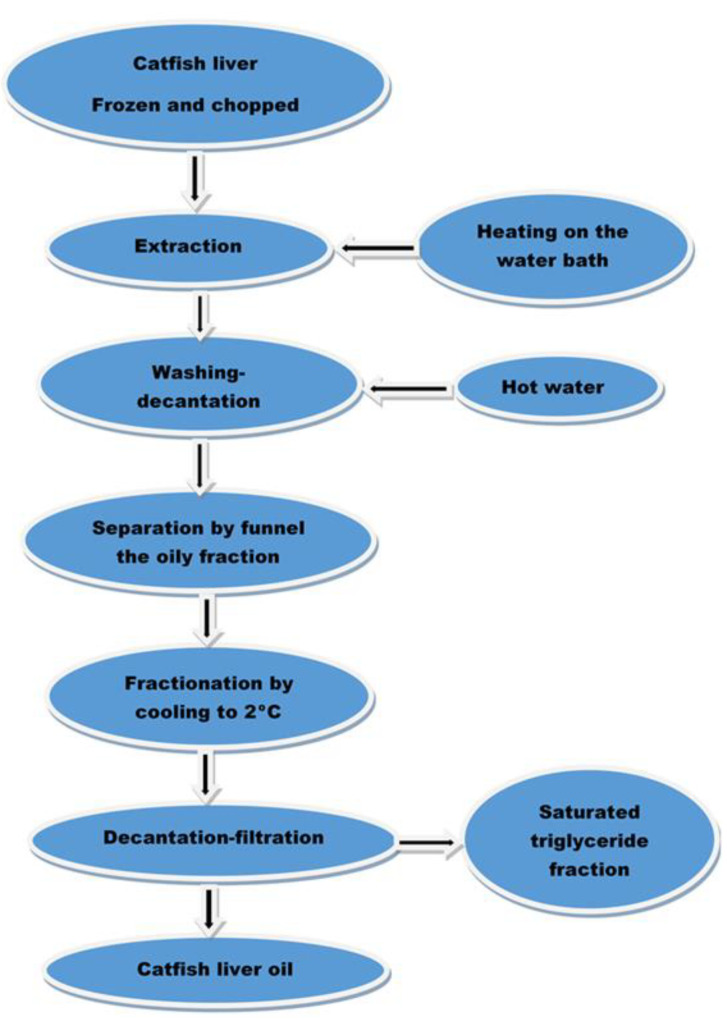
Technological scheme for obtaining stingray (catfish) liver oil.

Stingray liver taken from six wild specimens fished in the Black Sea in August 2022 was used for extraction (Figure 13). After washing, the liver was subjected to freezing at −20 °C. The frozen liver was minced and heated in a water bath. The obtained oily fraction was washed 2–3 times with warm distilled water using a separatory funnel, after which it was subjected to the fractionation operation by cooling to 2 °C when the saturated triglyceride fraction settled (Figure 14). The liquid oily part was separated by decantation and filtration from the sedimented triglyceride fraction. The oil thus obtained was stored in the dark and at low temperatures (8–15 °C).

### 4.2. Analysis of Black Sea Stingray (Dasyatis pastinaca) Liver Oil

The stingray liver oil was subjected to the following analyses: determination of the density at 20 °C, the refractive index, the acidity index, the iodine index, the saponification index, and the peroxide index according to the provisions of the Romanian Pharmacopoeia Edition X [56].

For the analysis of fatty acids from catfish liver oil, an internal standard (C 23:0 methyl ester; Nuchek Prep Inc., Elysian, M.N., USA) was added, and the mixture was dried under a nitrogen atmosphere and then subjected to hydrolysis using a 7.9% KOH solution in methanol. After cooling, the samples were treated with a 20% boron fluoride solution in methanol. Fatty acid methyl esters were detected with a gas chromatograph 17 Shimadzu GC (Kyoto, Japan) using helium as a carrier gas. The peak area was processed using Shimadzu Class GC-10 software (Version No., company name, Kyoto, Japan) [57].

**Figure 15:**
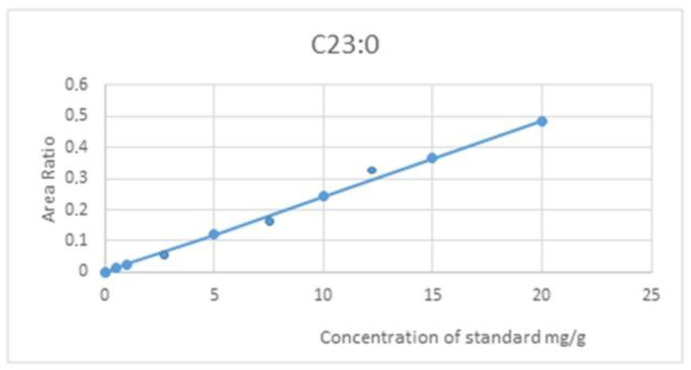
Calibration curve for the analysis of fatty acids in stingray liver oil (Y = 0.0243x + 0.0001).

All samples were tested three times, and the results were expressed as means ± SD (standard deviation). A standard mix of fatty acids and methyl esters (Nuchek Prep Inc., Elysian, MN, USA) was used to calibrate gas chromatography and determine response factors (Figure 15). All reagents used (Sigma-Aldrich, Schnelldorf, Germany) were of analytical purity.

### 4.3. Formulation of Ointment with Stingray (Dasyatis pastinaca) Liver Oil

The ointment with stingray liver oil was prepared according to the following formula: stingray liver oil (30 g), lanolin (15 g), and petroleum jelly (up to 100 g). The lanolin and petroleum jelly were liquefied in a water bath, then the stingray liver oil was added and homogenized until completely cooled.

Cosmetic lanolin was used (MAYAM brand). With an emollient and strong moisturizing action, active substances are transported into the skin to protect and repair dry, scaly, cracked, or itchy skin. This is a classic ingredient for pharmaceutical preparations, ointments, and traditional creams. Raw lanolin is anhydrous (does not contain water) and is obtained by processing sheep’s wool. It is secreted by the sebaceous glands in the sheep’s skin and is present on the wool threads. MAYAM lanolin is obtained from wool not affected by pesticides. The composition of petroleum jelly is a mixture of solid and liquid carbohydrates. Vaseline is obtained during the processing of petroleum fractions with a low boiling point, and its invention dates back to the middle of the 19th century. Petroleum jelly melts at 60 °C, dissolves in ether and chloroform, and is miscible with all oils except castor. At the same time, it does not dissolve in water or alcohol, so when applied to the skin, it washes off with difficulty. Cosmetic petroleum jelly is used in the manufacture of many ointments and creams. Cosmetic petroleum jelly from the Vaseline manufacturer was used for the preparation.

The ointment was subjected to additional analyses for characterization: appearance analysis, pH determination, thermal stability determination, viscosity determination, and spreadability determination.

The appearance was analyzed by a magnifying glass examination (4.5×) of a sample of ointment spread in a thin layer on a microscopic slide.

The determination of the pH was carried out after the processing of the preparations, namely the extraction of the samples with distilled water (1:5) and the measurement of the pH of the aqueous phase that separates after heating in a water bath at 60 °C and homogenization for 10 min. The Radelkis pH meter (Budapest, Hungary) was used to determine the pH.

The determination of the thermal stability was performed by keeping the samples in two temperature conditions (at 2 °C and at 40 °C for 8 h) as follows: 5 g of the sample from the ointment are introduced into weighing vials equipped with lids, and the vials are kept in the oven and in the refrigerator at the mentioned temperatures, after which the appearance of the samples, which must be kept homogeneous, is examined.

Viscosity was determined using a VEVOR NDJ-9S Digital Rotary Viscometer (Kansas City, USA).

The spreadability of the ointment was examined 28 h after preparation by measuring the spreading diameter of 1 g of sample placed between two 20 × 20 cm glass plates after 1 min using the Ojeda Arbussa method [58]. The upper plate mass was standardized at 125 g. The spreading areas reached by the sample were subsequently placed over the sample at 1 min intervals with weights of 50 g, 100 g, 150 g, 250 g, 500 g, and 750 g measured in millimeters. The determinations were repeated 30 days after the preparation of the ointment. The results were expressed in terms of the spreading area as a function of the applied mass according to the following equation:

S_i_ = d_i_^2^ (π/4) (1)

in which:

- Si is the spreading area (mm^2^) resulting from the applied mass “i” (g), and
- di is the mean diameter (mm) reached by the sample.

### 4.4. Testing the Healing Action of Stingray (Dasyatis pastinaca) Liver Oil Ointment

For this experiment, three groups of 10 male Wistar rats weighing 200 ± 10 g were used. The animals were kept in laboratory conditions for 2 days to get used to their new habitat (experimental room temperature of 22 ± 2 °C, humidity of 40–50%). The diet consisted of feeding at 8:00 and 17:00 and drinking water ad libitum from bottles.

Studies on laboratory animals were approved by the Scientific Research Ethics Commission of Carol Davila University of Medicine and Pharmacy, established under the Animal Protection (Code of Ethical Conduct 372/11.10.2022) Animal Welfare Act 1999 [59,60]. The clinical studies were carried out in compliance with the legislative norms.

The animals had shaved hair on their backs. After anesthesia with ether, wounds were produced by means of a device consisting of a metal disk with a diameter of 1 cm that was heated in water with 5% NaCl at 105 °C. The heated disk was applied to the dorsal shaved area and maintained for 10 s [61]. The animals were distributed by the randomization method into groups of 10 animals and were treated as follows:

Group 1—control group, untreated;

Group 2—the reference group treated with the cicatrizing ointment from the Romanian pharmaceutical market, Cicatrizin^®^, produced by the company Tis Farmaceutic S.A., which contains natural plant extracts in the composition (mallow, St. John’s wort, chamomile, and calendula);

Group 3—the tested group treated with the stingray liver oil ointment.

The treatment was applied twice a day for 12 days. Wound development was observed every two days by measuring the treated areas (in mm^2^) compared to those of the untreated control group and the Cicatrizin^®^-treated group (reference group). During the study, the clinical condition of the animals was monitored.

The healing effect (E%) was calculated according to the following formula:

E % = (A_i_ − A_i+1_/A_i_) × 100 (2)

where,

A_i_—the average wound surface at the initial moment (i);

A_i+1_—the average wound surface at the moment i + 1.

All determinations were performed in triplicate, and the results were expressed as mean ± SD (standard deviation). Statistical evaluation of clinical results was performed by Student’s t test (t test) and analysis of variance (ANOVA) [62,63].

### 4.5. Formulation of Emulgels with Stingray (Dasyatis pastinaca) Liver Oil

Four emulgel formulas were prepared according to the composition shown in Table 7.

Carbopol 940 (Merck, Darmstadt, Germany) was hydrated using purified water (conductivity below 0.05 µS/cm; obtained in a system SGW Ultraclear UV PlusTM, Darmstadt, Germany) provided in the formula (Table 1) for at least 24 h in the presence of glycerin (purity over 99%; Merck, Germany) used as a dispersing agent. Triethanolamine (purity over 99%; Triethanolamine from Carl Roth GmbH + Co. K.G., Germany) was used for neutralization at the end of this interval. Catfish liver oil was emulsified in a gel base in the presence of the emulsifying agent Tween 80 (Merck, Darmstadt, Germany).

The prepared emulgels were subjected to analyses to assess their stability: appearance analysis, pH determination, thermal stability determination, viscosity determination, and spreadability determination, according to the techniques presented in the analysis of the previously prepared fatty ointment.

The rheological characteristics were analyzed using the Multi-Visc Rheometer rotational viscometer (Fungilab, Maharashtra, India) through stationary shear analysis at 33 °C ± 0.1 °C (close to skin temperature). Maintaining the temperature during the analysis was performed using a ThermoHaake P5 Ultrathermostat (Huston, USA). The three emulgel samples taken in the analysis were sheared at a shear rate specific to the TR 10 standard spindle, from 0.08 to 16.8 s^−1^, corresponding to a rotational speed between 0.3 and 60 rpm. Upward rheograms of shear stress as a function of shear rate were obtained. The rheological data were further analyzed by applying different shear stress (τ) versus shear rate ($\dot{\gamma }$) models as follows: Casson (Equation (3)) and Herschel-Bulkley (Equation (4)):

(3) $$\tau^{0.5}=\tau_{0}{}^{0.5}+\eta^{0.5}\cdot {\dot{\gamma }}^{0.5}$$

(4) $$\tau =\tau_{0}+K\cdot {\dot{\gamma }}^{n}$$

where,

η is plastic viscosity (Pa·s), τ_0_ is yield stress (Pa), K is consistency index (Pa·s^n^), and n is flow index (dimensionless) [64,65]. The flow parameters were determined using Table Curve 2D software (Version No.5.01, Systat Software GmbH, Erkrath, Germany).

For internal morphology analysis, the emulgel samples were measured using a scanning electron microscope (SEM, Thermo Fisher Scientific, GmbH, Dreieich, Germany). SEM images were obtained using electron beam scanning at an accelerating voltage of 15 KV and a magnification of 1000×.

### 4.6. Testing the Anti-Inflammatory Action of Stingray (Dasyatis pastinaca) Liver Oil Emulgel

For this experiment, six groups of 10 male Wistar rats weighing 230 ± 15 g were used. The animals were kept in laboratory conditions for 2 days to get used to their new habitat (experimental room temperature of 22 ± 2 °C, humidity of 40–50%). The diet consisted of feeding at 8:00 and 17:00 and drinking water ad libitum from bottles.

The tests were carried out using two experimental methods of acute inflammation: the edema induced in the rat’s paw with a 10% kaolin suspension and the one with a 6% dextran solution.

By injecting kaolin into the rat’s paw, the formation of prostaglandins is stimulated, causing local inflammation and edema [66]. Dextran-induced edema is mainly due to the release of histamine and serotonin and is called anaphylactoid edema.

Edema was induced by the intra-plantar injection of 0.1 mL of 10% kaolin suspension and 0.2 mL of dextran solution.

For each edematous agent, three groups of 10 male Wistar rats were used. One group constituted the control group; one group was treated with the stingray liver oil emulgel formula C (the best formula according to the rheological evaluations); and one group was treated with Diclofenac gel (10 mg/g) produced by the Fiterman company. All animals were administered the edematous agent. On the paw in which the edema was induced, the test preparation was applied uniformly in a thin layer of ~0.25 g gel.

**Figure 16:**
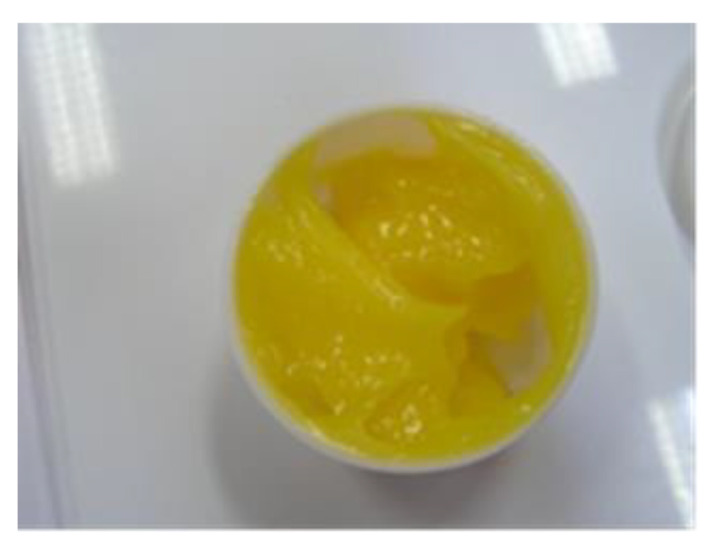
Stingray liver oil emulgel (Formula C).

Group 1—control group, untreated;

Group 2—the reference group treated with Diclofenac gel (10 mg/g) produced by the Fiterman company;

Group 3—the tested group treated with the catfish liver oil emulgel (Figure 16).

The evaluation of the anti-inflammatory effect of the ointment taken in the study was compared to the preparation of Diclofenac gel (10 mg/g produced by the Fiterman company) existing on the Romanian pharmaceutical market, applied to the paw with edema under the same conditions previously detailed.

The determinations were made against control groups (untreated individuals).

The volume of the rat’s paw was measured with a plethysmometer, Ugo Basile 7140 (Gemonio, Italy). After the intra-plantar injection of the edematous agent, further measurements were performed at intervals of 2 h, 4 h, 6 h, and 24 h (for the edematous agent, 10% kaolin suspension) and at intervals of 30 min, 60 min, 90 min, and 120 min from the induction of edema (for the edematous agent, 6% dextran solution).

The average value of anti-inflammatory edema (expressed in mL) and the percentage of edema inhibition effect for each batch were calculated according to the formula:

Edema inhibition effect (I) % = (X control − X treatment agent/X control) × 100(5)

where,

X treatment agent represents the average value of the edema produced by the tested gel (Diclofenac gel or emulgel Formula C);

X control represents the average value of the edema produced in the control group in the same time interval after the administration of the edematous agent.

## 5. Conclusions

Stingray liver oil obtained through a process that does not alter the bioactive compounds in its composition represents a resource with significant therapeutic potential in the context of its capitalization as a by-product obtained from catfish breeding farms that pursue the exploitation of the skin of the stingray in the leather goods industry. Similar to all lipids of marine origin, stingray liver oil is an important source of polyunsaturated fatty acids and can be used in the development of therapeutic formulas for internal or external use. Having a significant healing and anti-inflammatory action and a composition rich in valuable nutritional principles, stingray liver oil represents a valuable resource for the dermato-cosmetics industry of bioactive natural products. Clinical studies indicate an increased therapeutic potential of stingray liver oil and good versatility for its use in various dermato-cosmetic preparations.

## Figures and Tables

**Table 1:** Values of examined parameters relative to stingray liver oil.

Parameter	Value ± SD
Iodine value (g I_2_/100 g fatty acids)	111.85 ± 0.66
Acid value (mg KOH/g sample)	4.93 ± 0.33
Saponification value (mg KOH/g sample)	179.07 ± 0.25
Peroxide index (mEq O_2_/kg)	0.8 ± 0.55
Density at 20 °C (g/mL)	0.921 ± 0.33
Refractive index	1.479 ± 0.55

SD—standard deviation.

**Table 2:** Percent distribution of fatty acids in the total lipid extract isolated from stingray liver oil.

Fatty Acid	mg/g ± SD (%)
C 10:0	3.42 ± 0.23
C 12:0	0.06 ± 0.13
C 14:0	4.23 ± 0.45
C 14:1	2.12 ± 0.22
C 15:0	0.16 ± 0.16
C 16:0	10.15 ± 1.84
C 16:1	12.23 ± 1.52
C 16:1ω-7	6.12 ± 0.81
C 17:0	1.15± 0.12
C 17:1	0.23 ± 0.18
C 18:0	8.85 ± 0.65
C 18:1	15.46 ± 1.73
C 18:1ω-7	4.75 ± 0.33
C 18:1ω-9	9.81 ± 0.74
C 18:2ω-6	2.18 ± 0.52
C 18:3	1.15 ± 0.25
C 18:3ω-3	2.12 ± 0.14
C 20:1	0.19 ± 0.38
C 20:3	0.08 ± 0.04
C 20:4ω-6	2.68 ± 0.55
C 20:5ω-3	3.86 ± 0.28
C 22:1	0.07 ± 0.14
C 22:5ω-3	1.49 ± 0.16
C 22:6ω-3	6.44 ± 0.31
Σ saturated fatty acids	31.02
Σ ω-3	13.91
Σ ω-6	4.86
Σ monounsaturated fatty acids	29.53
Σ polyunsaturated fatty acids	39.45
ω-3/ω-6	3.68
Polyunsaturated fatty acids/Saturated fatty acids	1.27
Polyunsaturated fatty acids/Monounsaturated fatty acids	1.33

SD—standard deviation.

**Table 3:** Characteristics of catfish liver oil ointment.

Parameter	Result
Appearance	Homogeneous appearance, yellow-orange color, characteristic smell
pH	6.0
Thermal stability	Good stability, the samples remained homogeneous at the subjected temperature steps without separating into several phases
Viscosity	285 mPa/s

**Table 4:** Emulgels characteristics.

Characteristic	Formula A	Formula B	Formula C	Formula D
Initial macroscopic characteristics	appearance: homogenous; color: yellowish; smell: specific	appearance: homogenous; color: yellow; smell: specific	appearance: homogenous; color: orange-yellow; smell: specific	appearance: homogenous; color: orange-yellow; smell: specific
Macroscopic characteristics after 30 days	appearance: homogenous; color: yellowish; smell: specific	appearance: homogenous; color: yellow; smell: specific	appearance: homogenous; color: orange-yellow; smell: specific	appearance: homogenous; color: orange-yellow; smell: specific
Initial pH	6.4	6.1	5.8	5.6
pH after 30 days	6.2	6.0	5.7	5.6
Initial thermal stability	Good stability without a tendency to separate	Good stability without a tendency to separate	Good stability without a tendency to separate	Good stability without a tendency to separate
Thermal stability after 30 days	Good stability without a tendency to separate	Good stability without a tendency to separate	Good stability without a tendency to separate	Good stability without a tendency to separate

**Table 5:** Herschel-Bulkley model flow parameters obtained through stationary shear analysis at 33 °C.

Gel/Flow Parameters	Yield Stress (Pa) (τ_0_ - Pa)	Consistency Index (K - Pa·s^n^)	Flow Index (n)	Viscosity at 0.3 rpm (η_0.3_ - Pa·s) Initial	Viscosity at 0.3 rpm (η_0.3_ - Pa·s) after 30 Days
Formula A	27.318	21.081	0.35	499.200	482.100
Formula B	47.233	33.738	0.29	872.700	810.200
Formula C	77.154	66.884	0.27	553.000	432.800
Formula D	53.366	35.114	0.28	861.600	755.600

**Table 6:** The coefficient values of rheological models tested at 33 °C.

Emulgel/Rheological Model	Casson	Herschel-Bulkley
Formula A	0.832	0.992
Formula B	0.975	0.997
Formula C	0.919	0.995
Formula D	0.969	0.996

**Table 7:** Emulgel compositions.

Components	Formula A	Formula B	Formula C	Formula D
Carbopol 940	1 g	1 g	1 g	1 g
Glycerin	5 g	5 g	5 g	5 g
Triethanolamine	0.5 g	0.5 g	0.5 g	0.5 g
Tween 80	0.5 g	0.5 g	0.5 g	0.5 g
Stingray liver oil	3 g	5 g	7 g	10 g
Purified water	until 100 g	until 100 g	until 100 g	until 100 g

## Data Availability

There are no data available for this publication.
